# Nucleotide Variants of the BH4 Biosynthesis Pathway Gene *GCH1* and the Risk of Orofacial Clefts

**DOI:** 10.1007/s12035-015-9342-8

**Published:** 2015-07-28

**Authors:** Kamil K. Hozyasz, Adrianna Mostowska, Piotr Wójcicki, Agnieszka Lasota, Małgorzata Zadurska, Izabela Dunin-Wilczyńska, Paweł P. Jagodziński

**Affiliations:** 1 Department of Paediatrics, Institute of Mother and Child, 17a Kasprzaka Str., 01-211 Warsaw, Poland; 2 Department of Biochemistry and Molecular Biology, Poznan University of Medical Sciences, Poznan, Poland; 3 University Clinic of Medical Academy, Wroclaw, Poland; 4 Department of Jaw Orthopaedics, Medical University of Lublin, Lublin, Poland; 5 Department of Orthodontics, Institute of Dentistry, The Medical University of Warsaw, Warsaw, Poland

**Keywords:** NSCL/P, *GCH1*, Segawa’s disease, Neurotransmitters, Individual variability

## Abstract

A deficiency of GTP cyclohydrolase, encoded by the *GCH1* gene, results in two neurological diseases: hyperphenylalaninaemia type HPABH4B and DOPA-responsive dystonia. Genes involved in neurotransmitter metabolism and motor systems may contribute to palatogenesis. The purpose of the study was to analyse polymorphic variants of the *GCH1* gene as risk factors for non-syndromic cleft lip with or without cleft palate (NSCL/P). Genotyping of nine polymorphisms was conducted in a group of 281 NSCL/P patients and 574 controls. The *GCH1* variant rs17128077 was associated with a 1.7-fold higher risk for NSCL/P (95 %CI = 1.224–2.325; *p* = 0.001). We also found a significant correlation between the rs8004018 and rs17128050 variants and an increased risk of oral clefts (*p*_trend_ = 0.003 and 0.004, respectively). The best evidence of the global haplotype association was observed for rs17128050 and rs8004018 (*p*_corr_ = 0.0152). This study demonstrates that the risk of NSCL/P is associated with variants of the *GCH1* gene related to BH4 metabolism and provides some evidence of the relationships between morphological/functional shifts in the central nervous system and orofacial clefts.

Non-syndromic cleft lip with or without cleft palate (NSCL/P, OMIM 119530) is a common birth defect in humans, with an approximate prevalence of 4 to 13 per 10,000 live births, and is notable for its significant lifelong morbidity and complex aetiology with both genetic and environmental factors contributing to the condition [1–3]. Moreover, approximately 12 % of all embryos or foetuses aborted have an orofacial cleft [4]. Well-known candidate genes that are correlated with the risk of NSCL/P in various populations include *IRF6*, *VAX1*, *MAFB*, *NOG*, *ARGHAP29*, and the 8q24.21 locus (gene desert) [1, 2]. However, their nucleotide variants do not account for all observed NSCL/P cases, emphasising the need for identifying new genetic factors associated with orofacial clefts [2]. There is uncertainty about the individual variability in response to surgery and rehabilitation in NSCL/P-affected patients as well as the validity of the theories that have determined the selection of some techniques over others [5, 6]. Increasing evidence suggests that genetic loci involved in a pathological facial trait contribute significantly to the variation of normal facial morphology and may be associated with the differences in treatment responses of individual NSCL/P patients [7].

Several reports have provided evidence that brain morphology is fundamentally altered in at least some portion of the NSCL/P population [8, 9]. This co-occurrence reflects the intimate developmental relationship between the face and the brain. For several decades, it has been known that the ingestion of certain poisonous plants from the *Conium*, *Lupinus*, and *Nicotiana* genera, which contain piperidine alkaloids, during critical periods of gestation induces cleft palate in sheep, goats, cattle, and pigs [10]. Panter et al. [11] demonstrated that piperidine alkaloids induce a reduction in foetal movement that is directly responsible for abnormal palatogenesis in livestock. Gamma amino butyric acid (GABA) is synthesised from glutamate by glutamic acid decarboxylase (GAD). Vesicular inhibitory amino acid transporter (VIAAT) is responsible for the storage of GABA. Gamma amino butyric acid subunit beta-3 receptors are encoded by the *GABRB3* gene and are major inhibitory neurotransmitter receptors in the central nervous system [12, 13]. In 2010, a study using knockout mice revealed that cleft palate may be the result of alternations in GABA signalling, due to the loss of *Gad1* and *Viaat* gene functions within the central nervous system during embryogenesis, which clinically manifests as a loss of normal foetal movements [12]. The lack of the *Viaat* expression in the palate suggests that its function is not required in the palate for normal palatogenesis [12]. Interestingly, there is evidence for an association between single nucleotide polymorphisms (SNPs) in *GABRB3* and *GAD67*, encoding isoform of GAD and a risk for NSCL/P [11, 13, 14].

6(R)-l-erythro-5,6,7,8-tetrahydrobiopterin (BH4) is synthesised de novo from guanosine triphosphate (GTP) through the catalysis of three enzymatic reactions by GTP cyclohydrolase (rate-limiting step), 6-pyruvoytetrahydrobiopterin synthase, and sepiapterin reductase [15]. GTP cyclohydrolase deficiency results in two neurological disorders: hyperphenylalaninaemia with absent urinary neopterin and biopterin (HPABH4B) and dihydroxyphenylalanine (DOPA)-responsive dystonia (DYT5a, also known as Segawa’s disease) with reduced CSF levels of total biopterin (of which BH4 is the main component) and neopterin. DYT5a is caused by a particular impairment of nigrostriatal dopamine neurons that are different from those involved in Parkinson’s disease and is a childhood-onset or adolescent-onset form of dystonia [15]. Diurnal fluctuation of dystonia with increasing severity over the course of the day is a common feature [15, 16]. There is usually gradual generalisation of symptoms with age. Patients with DOPA-responsive dystonia have sufficient BH4 stores to metabolise a normal intake of phenylalanine [16]. A recent study conducted by Lupo et al. [17] suggested an association between a polymorphic variation of the *GCH1* gene (OMIM *2643), encoding GTP cyclohydrolase, and abnormal neural tube closure.

Such findings have prompted the search for oral cleft-associated variants of genes involved in neurotransmitter metabolism and motor systems. Therefore, the present study was designed to evaluate whether common nucleotide variations in the *GCH1* gene may contribute to the risk of NSCL/P in the Polish population.

## Material and Methods

### Patients and Controls

Peripheral blood samples from 281 unrelated subjects with NSCL/P were obtained from the Department of Paediatrics and Paediatric Surgery at the Institute of Mother and Child in Warsaw, from the Department of Plastic Surgery Specialist Medical Center in Polanica Zdroj, and from the Department of Jaw Orthopaedics at the Medical University of Lublin. Eligibility was ascertained from detailed medical records. The non-syndromic designation was based on a diagnosis of isolated CL/P with no other apparent cognitive and structural anomalies. The control group consisted of 574 healthy, non-related individuals with no family history of clefts or other major congenital structural anomalies, whom were mostly patients attending local primary care paediatricians and general practitioners. All participants were Caucasians of Polish origin. DNA was isolated from peripheral blood lymphocytes by a salting-out extraction procedure. The experiments were approved by the local Ethics Committee at the Poznan University of Medical Sciences. Written and oral consent were obtained from the legal guardians of all of the participants.

### Single Nucleotide Polymorphism Selection and Genotyping

Single nucleotide polymorphisms (SNPs) in the *GCH1* gene were identified from the related literature, from the dbSNP database (http://www.ncbi.nlm.nih.gov/projects/SNP/), and with the use of genome browsers of the International HapMap Project (http://www.hapmap.org/index.html.en) and 1000 genomes (http://www.1000genomes.org/). A final set of nine SNPs (Table 1) were selected based on the following criteria: location within protein coding or putative regulatory regions, haplotype-tagging properties, and minor allele frequency (MAF) of at least 10 % in the Caucasian population. The linkage disequilibrium (LD) patterns and the structure of haplotype blocks across each gene were determined using genotype data from the HapMap database and the Haploview 4.0 software package (http://www.broad.mit.edu/mpg/*haploview*/).

**Table 1:** Characteristics of polymorphisms analysed in the *GCH1* gene

rs no.	Chromosomal position^a^	SNP function	Alleles^b^	MAF
rs10483639	chr14:55306457	N/A	C/G (FWD)	0.19
rs841	chr14:55310492	UTR-3	C/T (REV)	0.18
rs4411417	chr14:55320563	intron	C/T (FWD)	0.19
rs17128050	chr14:55343879	intron	C/T (FWD)	0.11
rs8004018	chr14:55350696	intron	A/G (FWD)	0.11
rs3783641	chr14:55360139	intron	A/T (FWD)	0.19
rs3759664	chr14:55371579	nearGene-5	C/T (FWD)	0.17
rs8007267	chr14:55378991	N/A	C/T (FWD)	0.17
rs17128077	chr14:55386034	N/A	C/T (FWD)	0.12

*MAF* minor allele frequency calculated from the control samples, *FWD* forward, *REV* revers

^a^According to the February 2009 human reference sequence (GRCh37)

^b^According to the Single Nucleotide Polymorphism database (dbSNP), underline denotes the minor allele in the control samples

Genotyping of nucleotide variants rs841, rs17128050, rs8004018, and rs17128077 was carried out on the *LightCycler* 480 system (Roche Diagnostics, Mannheim, Germany) using pre-designed and custom TaqMan SNP Genotyping Assays according to the manufacturer’s instructions provided by Applied Biosystems (Applied Biosystems, Foster City, CA). The genotypes were called using the endpoint genotyping LightCycler 480 Software version 1.5. The genotyping of rs10483639, rs4411417, rs3759664, and rs8007267 was carried out by high-resolution melting curve analysis (*HRM*) on the *LightCycler* 480 system. The analysis of rs3783641 was conducted by the digestion of the amplified PCR products with the BsrI restriction enzyme (PCR-RFLP). Primer sequences and genotyping conditions are available on request. For quality control, approximately 10 % of the randomly chosen samples were re-genotyped. Samples that failed genotyping were not repeated and were removed from statistical calculations.

### Statistical Analysis

For each SNP, Hardy-Weinberg (HW) equilibrium was evaluated in both patients and controls by the chi-square (*χ* ^2^) test. Statistically significant deviation from *HW* expectations was interpreted as a *p* value < 0.05. The differences in allele and genotype frequencies between cases and controls were determined using standard *χ* ^2^ and Fisher exact tests. SNPs were tested for an association with NSCL/P using the Cochran-Armitage trend test. Odds ratios (ORs) with 95 % confidence intervals (95 %CIs) were used to assess the strength of the association. The dominant and recessive models were analysed. The Bonferroni correction was used to correct for multiple testing, and the *p* values < 0.0056 (0.05/9 SNPs) were interpreted as statistically significant. The haplotype-based association analysis was performed using PLINK v1.07 (http://pngu.mgh.harvard.edu/~purcell/plink/). The omnibus haplotype test (jointly estimating all haplotype effects at a location) for sliding windows of two to four SNPs across the gene was conducted using logistic regression. Significant *p* values were corrected using the 10,000-fold permutation test.

## Results

### Single-Marker Association Analysis

**Fig. 1:**
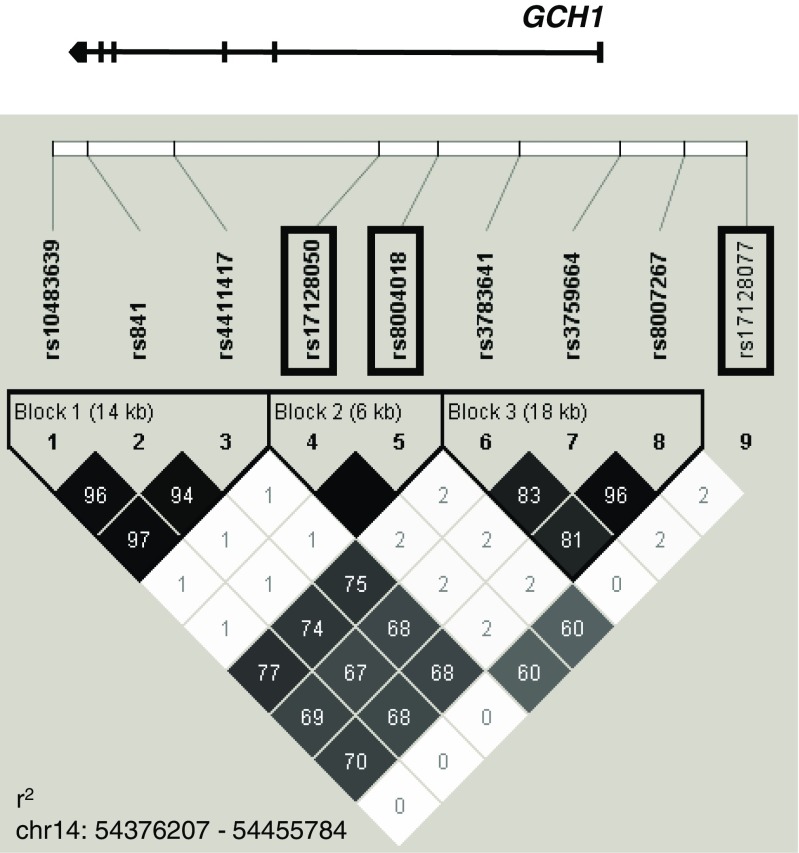
The linkage disequilibrium (LD) plot of SNPs analysed within the *GCH1* gene. The plot was generated using the genotype data from control samples and the Haploview 4.0 software (Broad Institute, Cambridge, MA). The names of SNPs significantly correlated with the risk of NSCL/P are enclosed in *boxes*. Numbers represent *r* ^2^ values expressed as a percentage of maximal value (1.0). *Squares without numbers* correspond to *r* ^2^ = 1.0. A *black-to-white gradient* shows highest (1.0) to lowest (0.0) *r* ^2^ value

None of the tested polymorphisms showed evidence of deviation from HW equilibrium in either cases or controls (*p* values > 0.05). The MAF for all SNPs was at least 11 %. The genotyping results, OR, and 95 %CI calculations for nine SNPs of the *GCH1* gene are reported in Table 2. Statistical analysis revealed a significant association between polymorphic variants of *GCH1* and an increased risk of NSCL/P (Table 2). The allelic and genotyping frequencies of rs17128077 were significantly different between NSCL/P patients and controls (*p*_genotypic_ = 0.005 and *p*_allelic_ = 0.001). The *p*_trend_ value for this SNP was 0.001. The OR for individuals with the rs17128077 T allele (TC or TT) compared to CC homozygotes was 1.687 (95 %CI = 1.224–2.325; *p* = 0.001). This result was statistically significant even after multiple testing corrections. Under the assumption of a dominant model, the calculated ORs for *GCH1* rs8004018 and rs17128050 were 1.604 (95 %CI = 1.154–2.229; *p* = 0.005) and 1.559 (95 %CI = 1.121–2.167; *p* = 0.008), respectively. These two SNPs were in complete LD with each other (*r* ^2^ = 1.00) and in moderate LD with rs17128077 (*r* ^2^ = 0.60) (Table 3, Fig. 1). For the remaining analysed *GCH1* SNPs, there was no evidence for both allelic and genotypic associations with the risk of oral clefts (Table 2).

**Table 2:** Association of polymorphisms of *GCH1* with the risk of NSCL/P

rs no.	Alleles^a^	MAF	Genotypes cases^b^	Genotypes controls^b^	*p*_trend_ value	*p*_genotypic_ value	*p*_allelic_ value	OR_dominant_ (95 %CI)^c^ *p* value	OR_recessive_ (95 %CI)^d^ *p* value
rs10483639	C/G	0.19	10/87/184	25/165/384	0.879	0.718	0.877	1.065 (0.788–1.440); 0.680	0.810 (0.384–1.712); 0.581
rs841	C/T	0.18	9/88/183	23/164/386	0.765	0.626	0.763	1.094 (0.809–1.480); 0.559	0.794 (0.362–1.740); 0.564
rs4411417	C/T	0.19	9/86/185	27/163/383	0.860	0.510	0.857	1.035 (0.765–1.401); 0.823	0.672 (0.311–1.448); 0.307
rs17128050	C/T	0.11	8/72/200	7/110/456	**0.004**	0.016	**0.004**	1.559 (1.121–2.167); 0.008	2.378 (0.853–6.627); 0.088
rs8004018	A/G	0.11	8/73/199	7/109/457	**0.003**	0.011	**0.002**	**1.604 (1.154–2.229); 0.005**	2.378 (0.853–6.627); 0.088
rs3783641	A/T	0.19	9/90/182	28/161/385	0.879	0.302	0.876	1.108 (0.821–1.496); 0.503	0.645 (0.300–1.387); 0.258
rs3759664	C/T	0.17	10/79/192	21/154/398	0.797	0.929	0.792	1.054 (0.775–1.434); 0.737	0.970 (0.450–2.089); 0.938
rs8007267	C/T	0.17	9/79/192	21/153/400	0.866	0.857	0.863	1.054 (0.774–1.435); 0.740	0.875 (0.395–1.936); 0.741
rs17128077	C/T	0.12	9/80/191	10/114/449	**0.001**	**0.005**	**0.001**	**1.687 (1.224–2.325); 0.001**	1.870 (0.751–4.656); 0.172

Statistically significant results (*p* value < 0.0056) are highlighted in bold font

*MAF* minor allele frequency calculated from the control samples

^a^Underline denotes the minor allele in the control samples

^b^The order of genotypes: dd/Dd/DD (d is the minor allele in the control samples)

^c^Dominant model: dd + Dd vs DD (d is the minor allele)

^d^Recessive model: dd vs Dd + DD (d is the minor allele)

**Table 3:** Linkage disequilibrium between polymorphisms of the *GCH1* gene in the control samples

	rs10483639	rs841	rs4411417	rs17128050	rs8004018	rs3783641	rs3759664	rs8007267	rs17128077
rs10483639		0.99	0.99	0.66	0.66	0.88	0.88	0.89	0.49
rs841	0.96		0.99	0.75	0.74	0.88	0.86	0.86	0.47
rs4411417	0.97	0.94		0.67	0.67	0.88	0.88	0.89	0.50
rs17128050	0.01	0.02	0.01		1.00	0.89	1.00	1.00	0.81
rs8004018	0.01	0.02	0.01	1.00		0.88	1.00	1.00	0.81
rs3783641	0.77	0.74	0.76	0.02	0.02		0.97	0.96	0.54
rs3759664	0.70	0.68	0.68	0.03	0.03	0.84		0.99	1.00
rs8007267	0.70	0.68	0.69	0.03	0.03	0.81	0.97		1.00
rs17128077	0.01	0.01	0.01	0.61	0.60	0.01	0.03	0.03	

D’ above diagonal, *r* ^2^ below diagonal

### Haplotype Association Analysis

Three two-SNP haplotypes (rs17128050-rs8004018, rs8004018-rs3783641, and rs8007267-rs17128077), one three-SNP haplotype (rs3759664-rs8007267-rs17128077), and two four-SNP haplotypes (rs10483639-rs841-rs4411417-rs17128050 and rs4411417-rs17128050-rs8004018-rs3783641) were associated with NSCL/P after permutation correction (Table 4). All of these haplotype combinations include nucleotide variants (rs17128050, rs8004018, and rs17128077) that were associated with the risk of NSCL/P in the single SNP association tests (Table 2). The best evidence of global haplotype association was observed for rs17128050 and rs8004018 (*p*_corr_ = 0.0152).

**Table 4:** Haplotype analysis of polymorphisms located in the *GCH1* gene

Number of SNPs	Haplotype combination	Chromosomal position^a^	*p* value	Corrected *p* value^b^
2	rs10483639_rs841	55306457–55310492	0.764	0.999
2	rs841_rs4411417	55310492–55320563	0.886	1.000
2	rs4411417_rs17128050	55320563–55343879	0.016	0.079
**2**	**rs17128050_rs8004018**	**55343879**–**55350696**	**0.003**	**0.015**
**2**	**rs8004018_rs3783641**	**55350696**–**55360139**	**0.008**	**0.039**
2	rs3783641_rs3759664	55360139–55371579	0.843	1.000
2	rs3759664_rs8007267	55371579–55378991	0.841	1.000
**2**	**rs8007267_rs17128077**	**55378991**–**55386034**	**0.005**	**0.025**
3	rs10483639_rs841_rs4411417	55306457–55320563	0.778	0.999
3	rs841_rs4411417_rs17128050	55310492–55343879	0.014	0.066
3	rs4411417_rs17128050_rs8004018	55320563–55350696	0.013	0.061
3	rs17128050_rs8004018_rs3783641	55343879–55360139	0.012	0.058
3	rs8004018_rs3783641_rs3759664	55350696–55371579	0.020	0.095
3	rs3783641_rs3759664_rs8007267	55360139–55378991	0.857	1.000
**3**	**rs3759664_rs8007267_rs17128077**	**55371579**–**55386034**	**0.006**	**0.028**
**4**	**rs10483639_rs841_rs4411417_rs17128050**	**55306457**–**55343879**	**0.010**	**0.045**
4	rs841_rs4411417_rs17128050_rs8004018	55310492–55350696	0.013	0.063
**4**	**rs4411417_rs17128050_rs8004018_rs3783641**	**55320563**–**55360139**	**0.010**	**0.049**
4	rs17128050_rs8004018_rs3783641_rs3759664	55343879–55371579	0.026	0.119
4	rs8004018_rs3783641_rs3759664_rs8007267	55350696–55378991	0.021	0.098
4	rs3783641_rs3759664_rs8007267_rs17128077	55360139–55386034	0.015	0.074

Statistically significant results are highlighted in bold font

^a^According to the February 2009 human reference sequence (GRCh37)

^b^ *p* value calculated using permutation test and a total of 10,000 permutations

## Discussion

Although great advances have been achieved in candidate gene identification for NSCL/P, the underlying molecular mechanisms remain obscure [1–3, 18]. Identifying the underlying aetiology is crucial for improving prevention strategies and genetic risk counselling. Recent experimental findings point toward at least some interaction between abnormal foetal movement and NSCL/P [12]. In this study, we assessed whether polymorphic variants in the *GCH1* gene, in which mutations may cause dystonia, are associated with NSCL/P in a sample from the Polish population. Our results suggest that the presence of the *GCH1* rs17128077 T allele is associated with a 1.7-fold increased risk for NSCL/P in the investigated population. We also found a significant correlation between the rs8004018 and rs17128050 variants with an increased risk for oral clefts. The association with single SNPs was confirmed at the haplotype level. We showed that two-, three-, and four-SNP haplotypes were associated with a risk for NSCL/P even after multiple testing corrections. To our knowledge, this is the first report evaluating the clinical impact of the *GCH1* rs17128077 variant. The interpretation of SNP results is especially challenging in complex disorders. It is unknown how these SNPs affect the gene expression level or the function of the encoded protein. In addition, there is no data showing the effect of analysed *GCH1* variants on the level of BH4 and total biopterins. Therefore, further in vitro and in vivo functional studies are needed to characterise the functional significance of these SNPs. The observed strong association between these SNPs and a risk for NSCL/P might be a result of complete or incomplete linkage disequilibrium with one or more yet-unknown functional variants of the *GCH1* gene. It is worth noting that the *GCH1* expression was detected in multipotent, proliferative human neural crest cells [19].

In 2006, Tegeder et al. [20] reported that among individuals with the haplotype consisting of the minor alleles rs10483639, rs3783641, and rs8007267 (C-A-T), the GCH1 mRNA levels were lower compared with other haplotypes. According to Zhang et al. [21], rs841 also affects the expression of *GCH1*. In addition, the excess production of BH4 is closely correlated with increased pain sensitivity [22–24]. In our study, the SNPs rs10483639, rs3783641, rs8007267, rs4411417, and rs841, which have been previously reported as conferring pain susceptibility, showed no association with the risk of NSCL/P [22–25]. The study conducted by Kim and Dionne [22] revealed no contribution of SNPs rs8004018 and rs17128050, rs8007267, rs3783641, rs841, rs10483639, or rs4411417 to pain sensitivity during the removal of the third molar teeth. The *GCH1* rs841 variant is reported to be associated with mildly increased blood pressure and heart rate [21] and serves as an independent predictor of deleterious long-term outcomes in ischemic stroke [26]. However, there are unfortunately no studies assessing the association between SNPs in the *GCH1* gene with pain or cardiovascular risk in the Polish population.

BH4 is an essential cofactor for three aromatic amino acid hydroxylases, including tyrosine hydroxylase (rate-limiting enzyme in dopamine biosynthesis), tryptophan hydroxylase (TPH2), and phenylalanine hydroxylase (PAH), as well as three isoforms of nitric oxide synthase (NOS), which catalyses the synthesis of nitric oxide from arginine, and alkylglycerol monooxygenase. Interestingly, in our previous studies, we showed that polymorphic variants of *PAH* and *TPH2* as well as haplotype combinations of the *TPH2* SNPs were significantly correlated with NSCL/P in the Polish population [27, 28]. Several studies have observed orofacial clefting and midfacial hypoplasia in offspring of women with untreated hyperphenylalaninemia [29]. In addition, abnormal results of orally administered phenylalanine loading tests in mothers of children with orofacial clefts and increased phenylalanine urine excretion in NSCL/P children were reported [30, 31]. Moreover, we have also demonstrated moderate evidence for the association of polymorphic variants of genes related to arginine metabolism (*ASS1*, *SLC25A13*) with abnormal palatogenesis [32]. The *NOS3* variants were reported to be associated with NSCL/P risk in a USA population [33].

Both the craniofacial region and neural tube arise from neural crest cells, leading to the hypothesis that there is a shared genetic background for orofacial clefts and spina bifida. In contrast to these expectations, in our study, the allele and genotype distributions of the three polymorphic variants of *GCH1* regarded as linked with spina bifida risk (rs8007267, rs3783641, rs10483639) [17] were found to be similar among NSCL/P affected children and controls. The haplotype rs8007267-rs3783641-rs10483639 was not associated with the risk of oral clefts; however, we identified three other haplotype combinations, including those variants separately that appeared to influence NSCL/P risk.

The *GCH1* gene has been studied in terms of its association with psychiatric disorders and cognitive function. Interestingly, an increased occurrence of autism has been observed in patients with NSCL/P [34]. Young children with autism demonstrate a reduced concentration of BH4 in cerebrospinal fluid, and treatment with this cofactor represents a novel therapy for autism spectrum disorders [35]. Schnetz-Boutaud et al. [36] genotyped four SNPs in *GCH1*, including one polymorphic variant analysed in our study (rs3783641), and observed no association in a dataset of 403 Caucasian families with autistic individuals. However, in this study, two SNPs of the gene encoding 6-pyruvoytetrahydrobiopterin synthase (*PTS*) showed a statistically significant association with autism [36]. In addition, there are also reports on the association of rs841 with personality traits: The rs841 CC homozygous male subjects had higher scores of novelty seeking, C allele carriers with chronic fatigue syndrome had higher scores for harm avoidance, and TT homozygotes had lower attention function [37].

We must note that the number of selected polymorphisms in the present study does not cover the complete *GCH1* gene. Allele frequencies are known to vary among different populations and different ethnic backgrounds. Because our study was conducted only in the Polish population, which is known to be ethnically homogenous, the results should be extrapolated to other ethnic groups cautiously. This study identified the association of polymorphisms with risk for NSCL/P without any attempt to establish a cause-and-effect relationship.

The *GCH1* gene appears to be highly mutable. To date, there are 138 reported different mutations/deletions associated with the phenotype DYT5a (http://www.biopku.org/biomdb/home.asp; Blau N, Thony B, accessed 6/11/2014). Most cases of Segawa’s disease present with the autosomal dominant form, but autosomal recessive mutations in *GCH1* have also been reported. The reason why such a variety of mutations occur in *GCH1* remains unknown. A wide range of neurological manifestations in patients with identical *GCH1* mutations is a well-known feature of DYT5a. It has been shown that NSCL/P is most often left-sided [1, 38]. It is noteworthy that DOPA-responsive dystonia during onset demonstrates a preference to the left side [16]. In addition, the coexistence of DOPA-unresponsive dystonia and cleft lip and palate in patients with Baraitser-Winter cerebrofrontofacial syndrome (BWCFF) was described [39]. BWCFF shows variable CNS involvement. Structurally, patients may have a normal brain or some degree of pachygyria with anteroposterior severity gradient [39]. The results of the present study, while by no means definitive, provide some evidence supporting earlier observations that orofacial clefts may be the result of alternations in the motor system during embryogenesis [12]. At present, the generalisability of these findings is uncertain, primarily due to the small sample size available and the lack of data on the neurological status of participants and their families. However, the complexity of embryogenesis leads us to hypothesise that a shift in the amount or the timing of expression of a single gene, such as *GCH1*, could affect multiple pathways and that the resulting phenotype may be influenced by common and rare genetic variants in this pathways.

In summary, we provide evidence for the role of *GCH1* nucleotide variants in the aetiology of NSCL/P in the Polish population. The traditional view has been that almost all behavioural deficits in individuals with NSCL/P are secondary manifestations of hearing and speech problems. The results of our study suggest that these deficits may stem also from direct relationships between morphological/functional shifts in the central nervous system and orofacial clefts. The factors suspected to be associated with the changes are polymorphic variants in the genes involved in neurotransmitters homeostasis and motor systems [11–14]. Additional association studies conducted in different populations as well as functional studies in appropriate model systems are needed to confirm our results and to explore the role of *GCH1* in craniofacial development.
